# Multi-hazard exposure mapping under climate crisis using random forest algorithm for the Kalimantan Islands, Indonesia

**DOI:** 10.1038/s41598-023-40106-8

**Published:** 2023-08-18

**Authors:** Sujung Heo, Sangjin Park, Dong Kun Lee

**Affiliations:** 1 Interdisciplinary Program and Life Science, Seoul National University, Seoul, Korea; 2 Korea Institute of Public Administration, Seoul, Korea

**Keywords:** Climate sciences, Environmental sciences, Natural hazards

## Abstract

Numerous natural disasters that threaten people’s lives and property occur in Indonesia. Climate change-induced temperature increases are expected to affect the frequency of natural hazards in the future and pose more risks. This study examines the consequences of droughts and forest fires on the Indonesian island of Kalimantan. We first create maps showing the eleven contributing factors that have the greatest impact on forest fires and droughts related to the climate, topography, anthropogenic, and vegetation. Next, we used RF to create single and multi-risk maps for forest fires and droughts in Kalimantan Island. Finally, using the Coupled Model Intercomparison Project (CMIP6) integrated evaluation model, a future climate scenario was applied to predict multiple risk maps for RCP-SSP2-4.5 and RCP-SSP5-8.5 in 2040–2059 and 2080–2099. The probability of a 22.6% drought and a 21.7% forest fire were anticipated to have an influence on the study’s findings, and 2.6% of the sites looked at were predicted to be affected by both hazards. Both RCP-SSP2-4.5 and RCP-SSP5-8.5 have an increase in these hazards projected for them. Researchers and stakeholders may use these findings to assess risks under various mitigation strategies and estimate the spatial behavior of such forest fire and drought occurrences.

## Introduction

Human interactions with natural extreme events, or hazards, are increasing globally^1^. Natural disasters have affected people and natural environments generating vast economic losses around the world^2^. Risks from hazard incorporate concepts of space, frequency, and scale as probabilities that occur within a specified period of time and within a potential damage area of a given size^2,3^. Most existing studies have focused on a single risk, even though risks have interactive properties^3–8^. Multi-hazard risk assistance can be useful in controlling these interactions.

Globally, droughts and forest fires are natural disasters that are increasing due to the climate crisis, causing serious damage such as human and ecological damage, economic damage, and forest damage^9^. In the case of Indonesia, many mountainous areas are experiencing illegal land conversion into farmland, in which artificial fires are occurring and government efforts are being thwarted by widespread damage^10,11^. The main affected areas were found to be concentrated in Sumatra and Kalimantan islands where Palm oil plantations were concentrated^12,13^. In 2019, forest fires broke out across Indonesia across Sumatra and Kalimantan. From 2015 to 2019, land was damaged by 317749.00 ha in central Kalimantan, 151919.00 ha in western Kalimantan, and 137848.00 ha in southern Kalimantan^14^. The peatland is recognized as the largest indigenous wetland area in Kalimantan and Sumatra regions. Due to the characteristics of peatland, it has a high moisture content and plays a crucial role during drought events^15^. Therefore, droughts in the peatland can act as contributing factors to increased forest fire risk. The reduction of moisture in the peatland due to drought can become a major cause of fire occurrence and its subsequent spread^16^.

In the event of an outbreak, such fires can cause problems with respiratory diseases in neighboring countries and residents through severe air quality pollution, as well as lead to drought and contribute to a serious climate crisis^17^. The fields of environment, culture, and public education were directly and significantly influenced^13^. Of greater significance, climate change leads to increased aridity, exacerbating drought severity and elevating the risk of forest fire occurrence^18^. Furthermore, the emission of carbon dioxide and greenhouse gases into the atmosphere resulting from forest fires contributes to the acceleration of climate change, which in turn increases the likelihood of more frequent and intense droughts and forest fires in the future^15^.

In previous studies, there have been studies on the potential disasters of forest fires, but no studies have provided complex disaster prediction and spatial analysis in conjunction with drought^19^. Sihombing^20^ evaluated the correlation and multiple risks of the two risk factors through a risk study on forest fires and earthquakes in Jakarta. While these studies exemplify multi-risk mapping, comprehensive studies of multi-risk assessment by machine learning models still lack examples of ring of fire countries. The development of multiple risk mapping approaches using new methods is critical to effectively managing risks in some areas^21–23^. Maps that depict droughts and forest fires risk have become more accurate recently because to GIS and RS technologies. Frequency ratio, logistic regression, weights of evidence, fuzzy reasoning, artificial neural networks, decision trees, support vector machines (SVM), and random forest (RF) models are a few examples of techniques. The RF model was chosen for this investigation since it is a relatively quick machine learning technique.

During the forest-building stages, it generates an accurate classifier with an internal, impartial generalizability estimate. It has a strong prediction performance and makes no statistical assumptions^24^. In the previous literatures, many researches have previously conducted hazard risk spatial analysis using ensemble models such as Support Vector Machine (SVM), Naive Bayes Classifier (NB), k-Nearest Neighbor (kNN), and Decision Tree (DT). Among them, the results from RF were found to be the highest^22,25,26^. Additionally, high AUC values have been observed in studies that evaluated hazards using RF alone^23,24,27^. Synthesizing these results, RF has been demonstrated as effective, and in addition to its good prediction performance, RF has been considered to measure the contribution of each variable to the occurrence of hazards and identify variables that play important roles in prediction^28^.

Tropical forests in Indonesia are among the planet’s most biodiverse, resource-rich ecosystems, but they are also among the most vulnerable to climate change and human-caused change in the future^29,30^. In addition to having a variety of cultural and traditional treasures, as well as biodiversity and ecosystems, Indonesia is renowned for having a variety of natural catastrophes, ranging in severity from mild to severe. Looking at the risk of minor hazard impacts, expression of national identity, position in the center of Indonesia, energy supply, protection from big disasters, etc., there are a number of justifiable arguments for the relocation of Indonesia’s new capital^31^. Indonesia intends to relocate its capital from Jakarta on the island of Java to Nusantara, a new city in a jungle on the island of Borneo.

Along with this, the city of Jakarta is sinking due to extensive groundwater extraction from underground aquifers. The administration of President Joko Widodo, often known as Jokowi, is now moving forward with a new project that is expected to cost roughly US $35 billion^32^. The East Kalimantan province of Borneo will become home to Indonesia’s capital city, which is now located in Jakarta on the island of Java. The city was then given the Sanskrit name Nusantara, which roughly translates to “archipelago” in English. Research that examines probable disasters in Indonesia’s future capital and its mitigation initiatives is thus absolutely important. Additionally, although Kalimantan exceeded Sumatra’s value in Indonesia’s total burned area in 2019, the Kalimantan bushfire case received less attention from several national media and central governments and the risk was not fully emphasized^14^. According to a thorough examination of the literature and to the best of our knowledge, there hasn’t been any study done yet on the multi-hazard modeling of forest fires and droughts in Indonesia. This study is significant since it is Indonesia’s first investigation of multi-hazard risk.

Indonesia has been severely damaged by natural disasters such as the Tsunami induced by earthquake, and East Kalimantan has been selected as a safer area. Here, then, I wondered what the expected damage from other disasters would be except for Tsunami and the earthquake on the island where the capital would be relocated. In this study, therefore, two major natural events (droughts and forest fires) in five provinces, Kalimantan Islands provide the basis for a multi-hazard risk assessment map. The main objective is to provide a useful and broad range of accurate multi-hazard mappings applicable to land use managers and other related stakeholders and to compare and predict the risks of each province within the study area.

In this study, multi-hazard (MH) risk were predicted and evaluated on Kalimantan Island. (1) Identifying the contributing factors that affect the occurrence of natural hazards; (2) Using Machine learning algorithm (RF) to create forest fire and drought risk probability maps; and (3) Estimating future drought and forest fire risks of the forecasted temperature and rainfall on representative concentration pathway (RCP-SSP) climate change scenarios and regional climate models.

Our work helps to create and evaluate machine learning techniques for mapping natural hazard-prone areas. By considering areas at risk from many risks, land use planners and policymakers can better understand the effects of the region’s urbanization process and implement mitigation strategies.

### Study area

**Figure 1:**
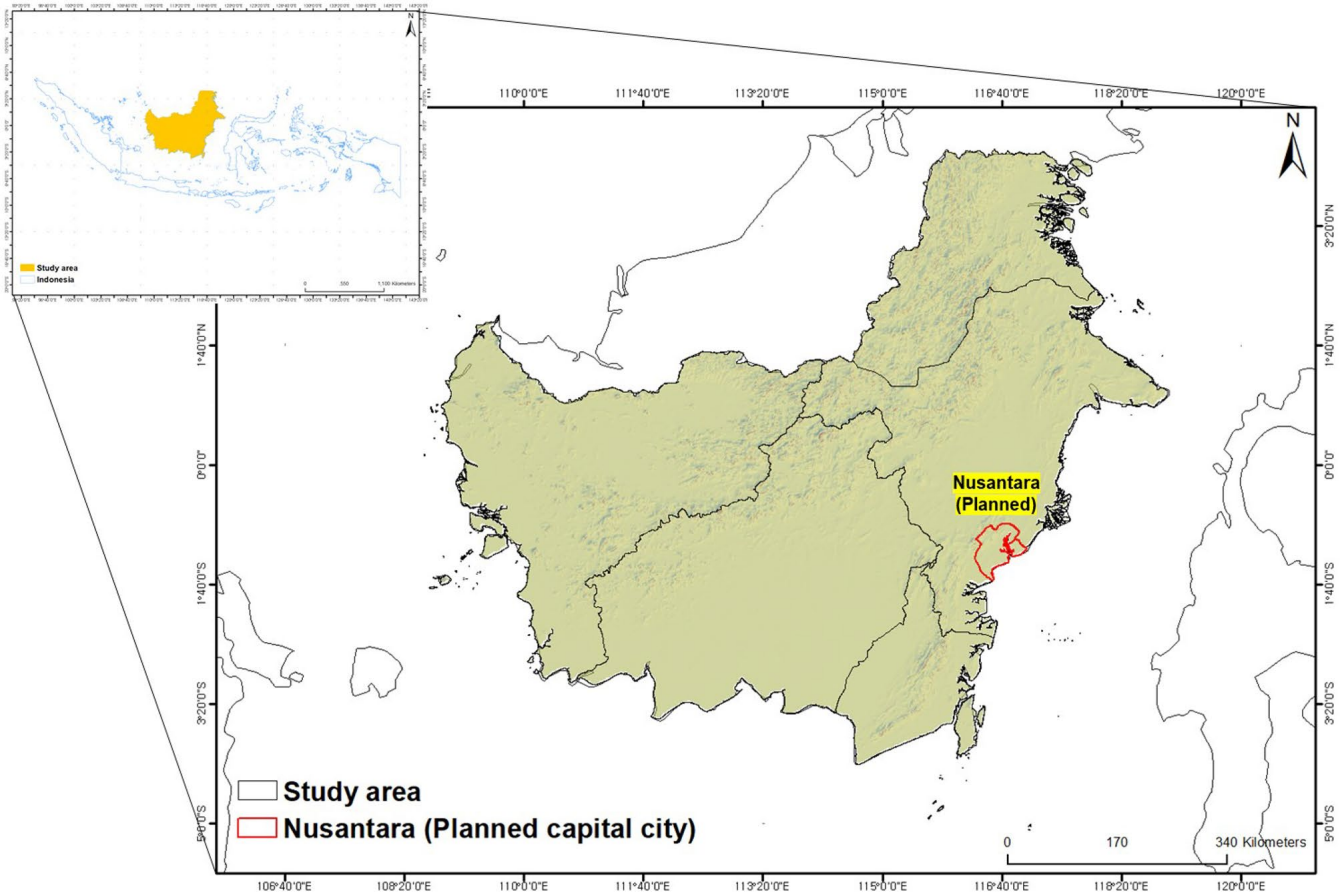
Study area (Kalimantan island).

With an area of around 539,238 km^2^, the research region is in Kalimantan between 1°S latitudes (Fig. 1). Kalimantan is now divided into five provinces—East Kalimantan, South Kalimantan, West Kalimantan, Central Kalimantan, and North Kalimantan. 73% of the island’s total area and 69.5% of its population (16,625,796 in the 2020 Census) are in Indonesian territory. The highest elevation is 4095 m above sea level and the mean elevation in the province is 104.9 m. The population of the islands is 23,053,723. Indonesia’s state is located in the Ring of Fire and a bill was passed in 2022 to move the capital from Java to Kalimantan Island due to typhoons, tsunami, and earthquakes.

### Methodology

**Figure 2:**
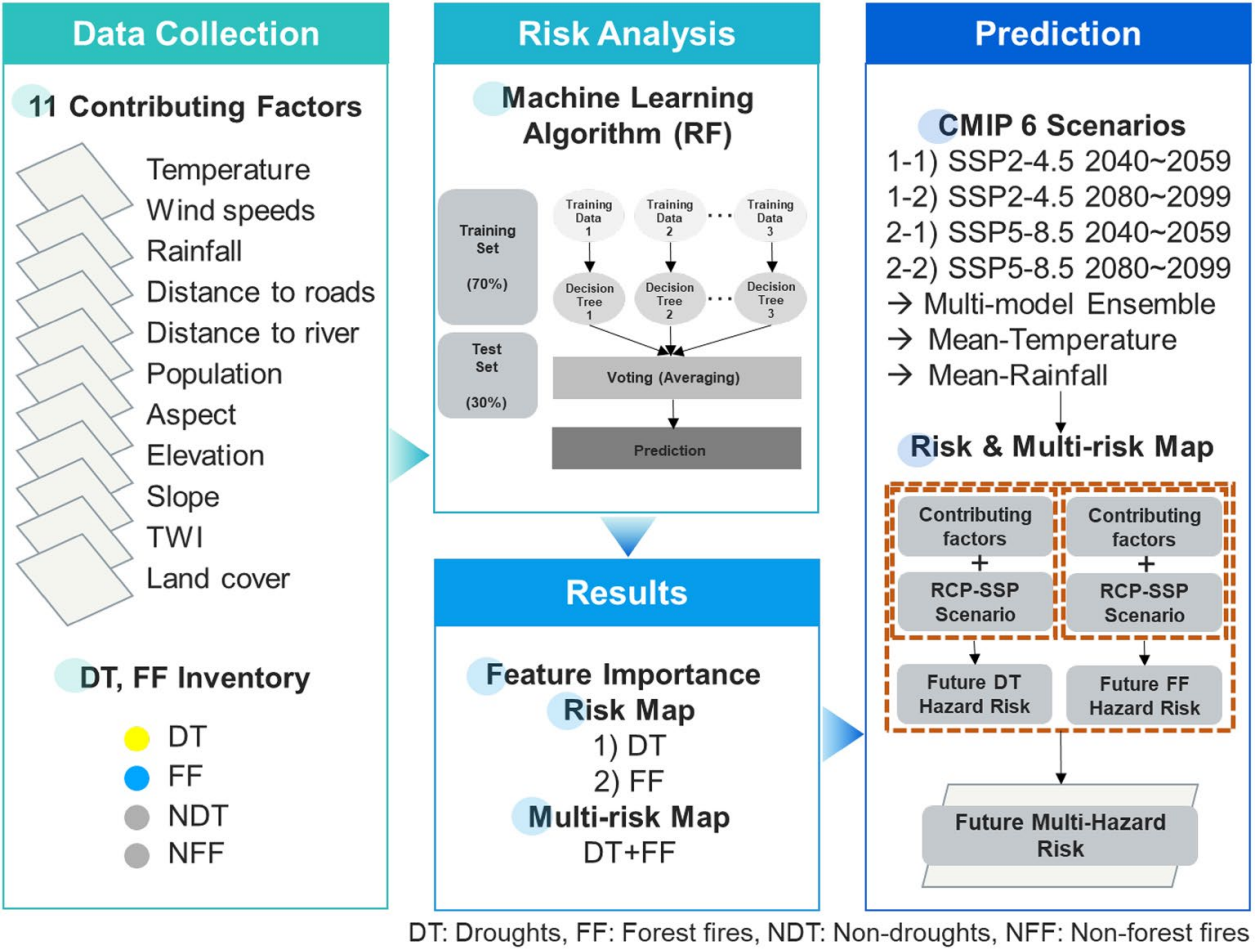
Framework of the study.

This study included three main activities (Fig. 2). (1) Collecting data over the last five years (2014–2019) at the study site; (2) Identifying the most important effective factors for each risk through literature review; (3) Building risk modeling and MH risk maps using machine learning algorithm (RF); (4) Applying RCP-SSP future climate scenarios.

### Hazards inventory data

**Figure 3:**
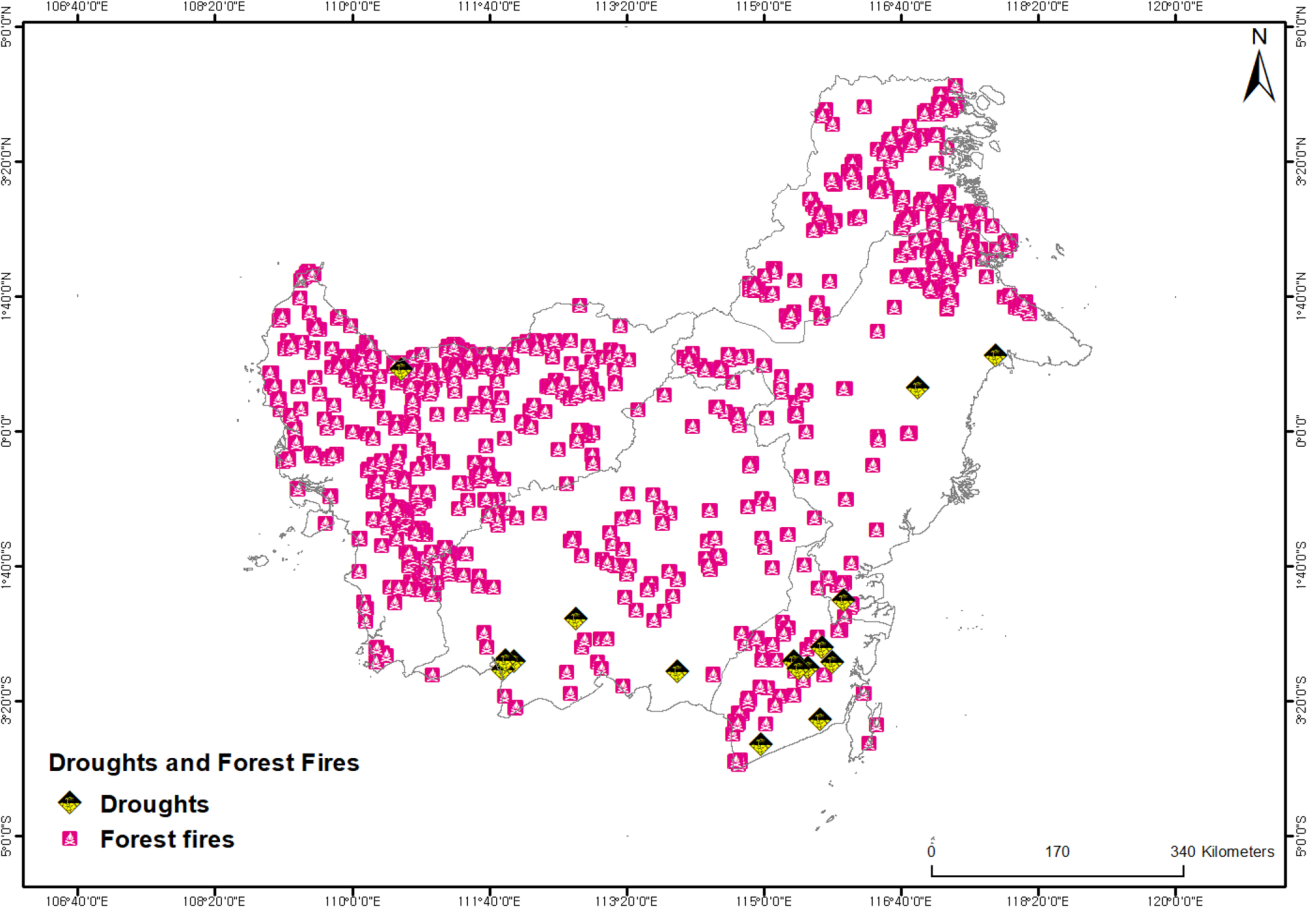
Forest fires and droughts inventory map of the study area.

This research located 1653 points that represent the sites of five different kinds of extremely dangerous occurrences that happened throughout the entire Kalimantan Islands over a five-year span^33^ (Fig. 3, Table 1). There were 16 droughts and 1637 forest fires among these occurrences. In the study area, it was observed that forest fires occur more frequently than droughts, and the anticipated major causes are as follows. Firstly, the El Niño phenomenon in the tropical Pacific region leads to elevated sea surface temperatures, resulting in prolonged periods of dryness. During such periods, accumulated dry vegetation, combustible grass, and dry leaves become highly susceptible to ignition^34^. Secondly, forest fires can be triggered by human activities such as illegal logging, the use of firearms, and improper disposal of cigarettes^35^. Lastly, the lack of effective forest management practices and the absence of adequate forest fire prevention and firefighting systems can also contribute to the spread of forest fires^11^. Data from both hazardous and non-hazardous locations were needed by the machine learning models in this research to perform modeling^36^. To balance the danger areas, three times as many non-hazardous places were randomly selected. Two sets of examples were created: one for training (70%) and the other for validation (30%).

**Table 1:** Data sources for forest fires and droughts risk maps.

Hazards	Sources	Type	Periods
Droughts	https://dibi.bnpb.go.id^37^	Converted to points from coordinates information	2014~2019
Forest fires	https://www.indonesia-geospasial.com/2020/04/shapefile-shp-titik-api-hotspot.html^33^	Points	2014~2019

#### Data collection of the contributing factors for forest fires and droughts

Effective factors for each hazard were measured and plotted in raster layers of 1 1 km pixel size in ArcGIS 10.8.2 based on a study of prior studies as well as a collection of recommendations from experts. The four groups of beneficial variables (Table 2) were climatic (temperature, wind speed, rainfall), anthropogenic (distance to roadways, people, distance to waterway), topography (slope, aspect, elevation, and topographic wetness index), and vegetation (land use). Given its many advantages, RF model is the best choice for the investigation’s approach. It makes no statistical assumptions and has a good prediction performance^24^. In contrast to other classification methods, trained classifier RF, which comprises of multiple decision trees, has a low error rate. The predictor with the greatest influence on the predicting function in relation to the other components will be the top splitter in each tree. As a result, every tree will be connected and have a similar structure (Supplementary Fig. 1).

**Table 2:** Contributing factors’ source for the forest fire and drought risk map.

Category	Factors	Sources	Datatype (resolution)	References
Climatic	Temperature	https://www.worldclim.org	Raster (1 km × 1 km)	^6,8,9,23,24^
	Wind speed			^2,23^
	Rainfall			^6,22,38,39^
Anthropogenic	Distance to road	https://www.diva-gis.org/gdata	Line	^2,22,24^
	Population	https://hub.worldpop.org/geodata/summary?id=29734	Raster (1 km × 1 km)	^2,8,23,24^
	Distance to river	https://www.diva-gis.org/gdata	Line	^2,24^
Topographic	Aspect	Calculated from DEM	Polygon	^2,22,24,39^
	Slope			^2,22,24,38,39^
	Elevation			^22,39^
	Topographic wetness Index			^2,39^
Vegetation	Land cover	https://www.indonesia-geospasial.com/p/sitemap.html		^2,22,24,38,39^

#### Multi-hazards risk mapping

Maps of forest fire (FF) and drought (DT) were produced using the machine learning algorithm and the contributing variables (Fig. 2). First, a RF model with a high Area under the receiver operating characteristic (ROC) Curve (AUC) value was used to generate vulnerability to each risk based on the dependent variables (locations of forest fires and droughts) and some environmental factors. Five classes—very low, low, intermediate, high, and very high—were used to classify the maps. A study of the literature^24^ revealed that high and very high susceptibility classes were considered high hazard (1) circumstances, while very low, low, and very low susceptibility classes were considered low hazard (0) conditions. The five-class maps created for each danger were transferred to these two classes: 0 and 1, to aid in integration. ArcGIS was used to merge the maps of forest fires and droughts to produce an integrated MH map, which was then categorized.

#### Accuracy assessment

Using the training group data for the goodness-of-fit test and the validation group data for the predictive-performance test, the precision of the MH maps was evaluated (AUC). AUC is a numerical measure and a technique without regard to thresholds^40^. A model’s categorization of a location is ideal when the area is 1, while a model’s classification of a location is poor when the area is 0.5 or less^25^. In the current research, Python 3.9 was used to create MH probability maps of woodland fires and droughts.

#### Future climate scenario application (RCP-SSP2-4.5 and RCP-SSP5-8.5)

Future climate scenarios (RCP-SSP2-4.5 and RCP-SSP5-8.5) were used to predict future risk potentials. In the previous studies, we found that the two scenarios were compared and reviewed^25,41–44^. In general, 2–4.5 are known realistic scenarios in which climate change is mitigated in the future with carbon dioxide reduction policies. On the other hand, 5–8.5 is the worst-case scenario in which there is almost no separate reduction policy and support^45^. We expected that the importance of the policy and the awareness of carbon dioxide reduction can be raised by comparing the two scenarios. Continuous variables (temperature and rainfall) were used to predict future risks from the effects of climate change. Bioclim variables from the 2040–2059s and 2080–2099s are built using the future climate conditions of the world data set expected under the IPCC 5th Report (CMIP6) (https://esgf-node.llnl.gov/search/cmip6/)’s Representative Concentration Pathways (RCP). The main task of Coupled Model Intercomparison Project’s Phase 6 (CMIP6) is the Scenario Model Intercomparison Project (ScenarioMIP), which will generate multi-model climate projections based on various scenarios of future emissions and land use changes using integrated assessment models^45^. RF classifier is built similarly to the method used in drought and forest fire risk analysis. In addition, to eliminate uncertainty, the process was carried out 1000 times against forest fires and drought risks.

## Results

### Accuracy assessments of the hazard maps using AUC

It was clear from evaluating the machine learning models’ accuracy (Table 3) that RF offered very accurate models. This model’s AUC values were all higher than 0.8, which indicated robust classification success and demonstrated the model’s tolerably high level of accuracy.

**Table 3:** AUC values for the RF model for visualizing forest fires and droughts.

Hazard type	AUC values
Droughts	0.875
Forest fires	0.880

### Feature importance

**Figure 4:**
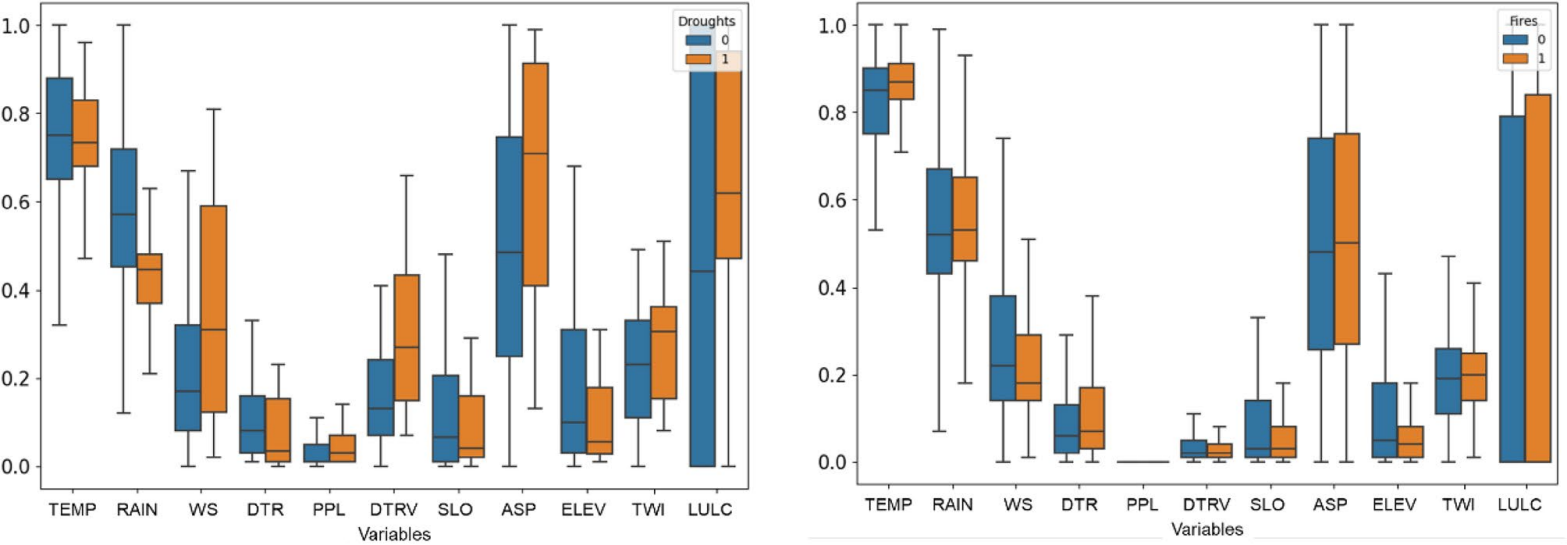
Risk map factor importance plot of droughts and forest fires using RF (*TEMP* Temperature, *WS* Wind speed, *RAIN* Rainfall, *DTR* Distance to road, *PPL* Population, *DTRV* Distance to river, *SLO* Slope, *ASP* Aspect, *ELEV* Elevation, *TWI* Topographic Wetness Index, *LULC* Land use and land cover).

It is important to understand how each contributing factor affects drought and forest fire risks and to assess the importance of each factor^24^. Figure 4 shows the importance of 11 variables selected for drought and forest fire risks using RF. To analyze this, we first divided the raw data used for risk analysis into drought and forest fires occurrence. Subsequently, each variable was normalized from 0 to 1, and the results were compared. It was observed that the aspect (ASP) had higher differences in normalized values for drought occurrences, while wind speed (WS) showed greater variations for forest fires compared to other variables. Therefore, it can be inferred that these factors are key elements when compared to other variables in each disaster. Nevertheless, it is important to note that these rankings may vary depending on the specific machine learning algorithm employed^46^.

### Integrated MH map

**Figure 5:**
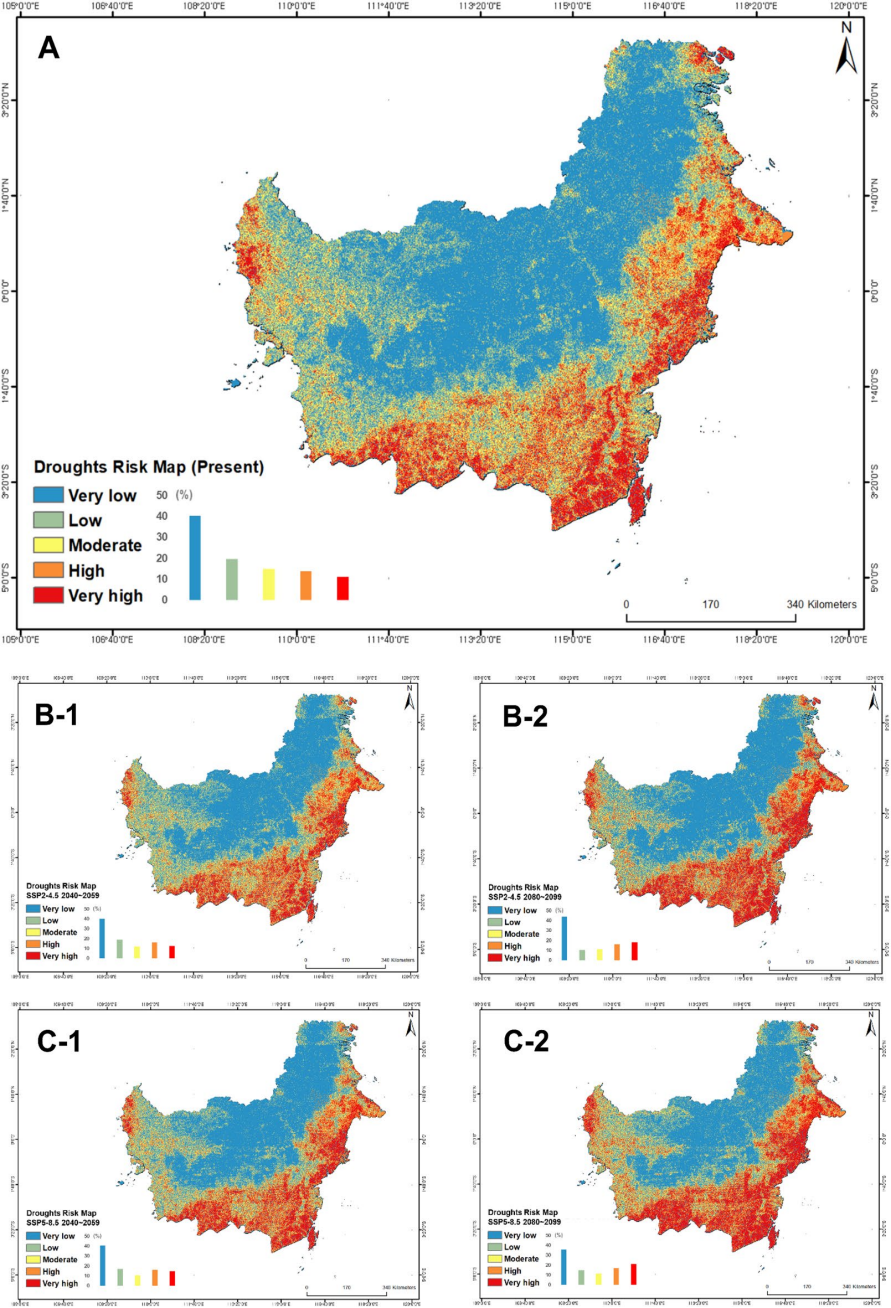
The risk map of droughts produced using RF model (*A* Present, *B-1* RCP-SSP 2–4.5 2040~2059, *B-2* RCP-SSP 2–4.5 2080~2099, *C-1* RCP-SSP 5–8.5 2040~2059, *C-2* RCP-SSP 5–8.5 2080~2099).

**Figure 6:**
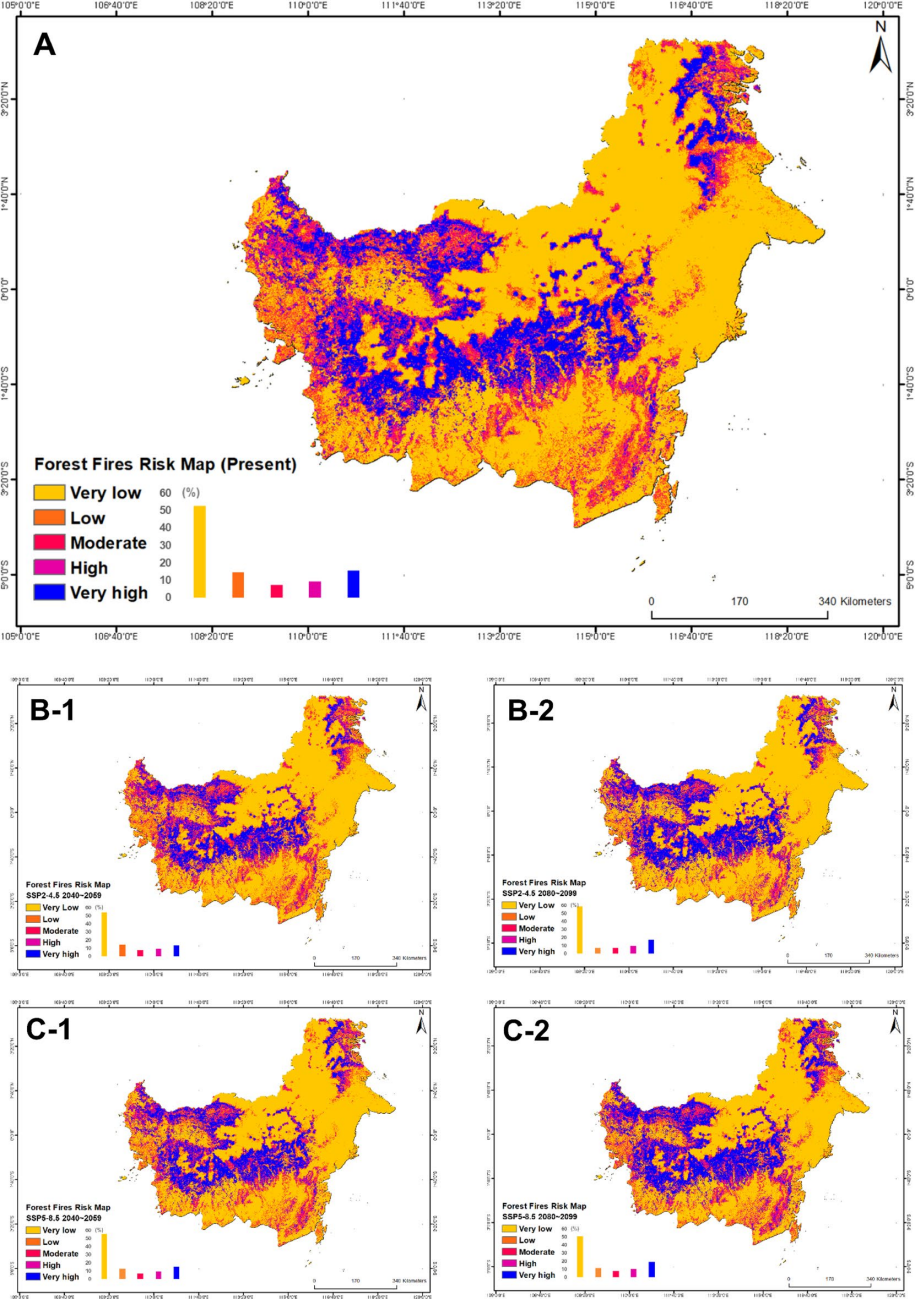
The risk map of forest fires produced using RF model (*A* Present, *B-1* RCP-SSP 2–4.5 2040~2059, *B-2* RCP-SSP 2–4.5 2080~2099, *C-1* RCP-SSP 5–8.5 2040~2059, *C-2* RCP-SSP 5–8.5 2080~2099).

Figures 5 and 6 show the risk probability map for droughts and forest fires. Specific risk percentages are described in Table 4. Both hazards’ risk-predicted areas were expected to increase in future scenarios rather than in the present. The MH map of the two disasters is shown in Fig. 6, and we found that both the risk of each disaster and the risk of connection increased in the future scenario.

**Table 4:** Risk values and ratio in forest fires and droughts risk map by RF.

Hazard	Scenario	Risk area (%)				
		Very low	Low	Moderate	High	Very high
Droughts	Present	40.2	19.7	15.0	13.9	11.2
	2–4.5 2050	40.4	18.8	12.2	16.3	12.4
	2–4.5 2080	43.9	10.3	11.4	16.3	18.1
	5–8.5 2050	40.9	17.3	10.7	16.3	14.9
	5–8.5 2080	35.7	14.7	11.6	17.2	20.9
Forest fires	Present	52.7	14.7	7.5	9.3	15.8
	2–4.5 2050	54.2	14.6	7.9	9.8	13.6
	2–4.5 2080	58.5	7.2	7.3	9.8	17.2
	5–8.5 2050	55.8	12.5	6.7	9.7	15.3
	5–8.5 2080	51.0	11.5	8.1	10.2	19.1

According to the results of the study, 53.13% of the sites are currently expected to be risk-free or have a low potential risk. However, 46.87% of the remaining research fields are expected to have at least one risk of drought or forest fire disaster. However, according to RCP-SSP2-4.5, these risks are expected to increase to 49.24% in 2040~2059 and 52.43% in 2080~2099, and to 52.43% in 2040~2059 and 60.02% in 2080~2099 on RCP-SSP5-8.5. Among them, the possibility of simultaneous forest fires and drought increased from 2.57% in present to 2.74% in 2040~2059 and 3.77% in 2080~2099 on RCP-SSP2-4.5, and to 3.77% in 2040~2059 and 6.42% in 2080~2099 on RCP-SSP5-8.5. Divided into five states, South Kalimantan had the highest risk of drought at 67.25%, West Kalimantan had the highest value of forest fires at 33.02%. Finally, South Kalimantan had the highest value of MH risks at 7.10%.

**Figure 7:**
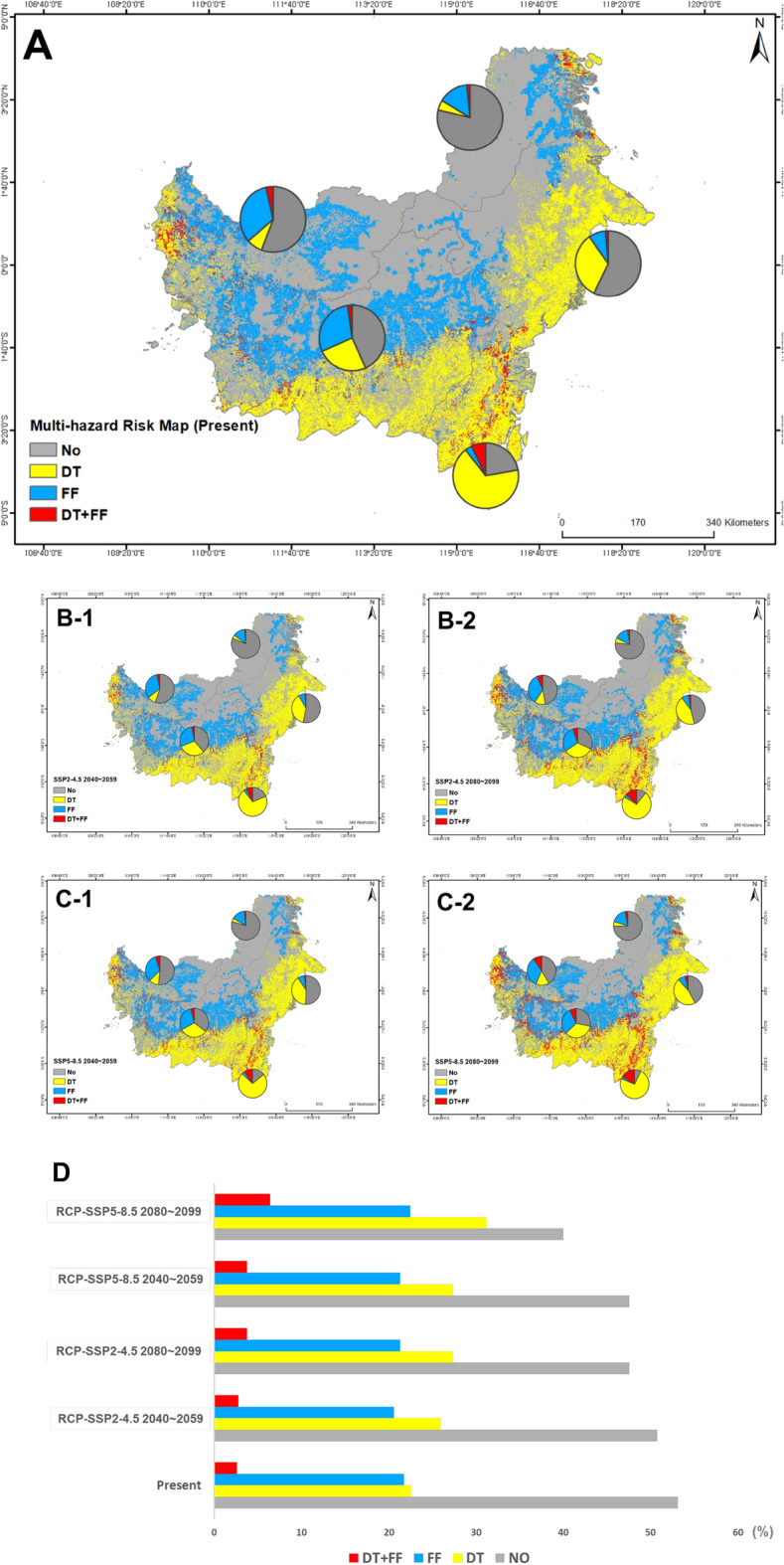
MH risk map (*No* Non or low risk; *DT* Droughts risk area; *FR* Forest fires risk area; and *DT* + *FR* Droughts and Forest fires risk area) and Future climate prediction (*A* Present, *B-1* RCP-SSP 2–4.5 2040~2059, *B-2* RCP-SSP 2–4.5 2080~2099, *C-1* RCP-SSP 5–8.5 2040~2059, *C-2* RCP-SSP 5–8.5 2080~2099, and *D* Percentage by hazard type and scenarios).

By region, the risk of forest fires was predicted to be greater in North Kalimantan and West Kalimantan than in drought in the current and future climate scenarios (Fig. 7). On the other hand, in East Kalimantan and South Kalimantan, drought will be higher than forest fire risk, and in Central Kalimantan, both disasters will have similar risk levels. Finally, South Kalimantan was the highest place where the two disasters occurred in conjunction, followed by West Kalimantan and Central Kalimantan.

## Discussion

We have created both a single-risk map and a MH risk map of drought and forest fires on Kalimantan Island, Indonesia, using machine learning model techniques. In addition, the sensitivity ratio to a specific disaster was separately indicated by dividing it by states.

According to the literature review, RF models perform better than other models in a variety of risk vulnerabilities than SVM and multivariate statistical analysis^25,47,48^. Most of this assessment suggested that creating several risk maps using optimal algorithm that deliver high accuracy and prediction performance of a single risk type can help to gain highly accurate information regarding relevance and interaction^38^.

Results show that predicting natural disasters with machine learning algorithm provides valuable data on how those hazards combine. Relationships between multiple dangers rely greatly on the scope of the study and the particular sets of hazards. In a MH research, Pourghasemi et al.^24^ used machine learning methods to map both the individual and group risks presented by three hazards (floods, forest fires, and landslides). Others have carried out MH risk evaluations, but one for each risk individually. A significant problem is figuring out how various hazards relate to one another and connect. This research starts to close that disparity. Furthermore, this study aligned with previous research where Random Forest (RF) demonstrated superior performance, employing supervised classification using spatial and geographic data, akin to findings by earlier researchers^49–51^. One of the distinguishing factors from prior studies lies in conducting spatial risk analysis of individual hazards and compound disasters through future climate scenarios^25,52^. Despite the inherent uncertainties of future climate scenarios, various scenarios from AR6 were employed, and the impacts based on policy scenarios were analyzed^53,54^. Most notably, this study incorporates the proposed new capital city of Indonesia, providing essential information and guidance for future urban planning in addressing climate change. The findings can serve as a tool to enhance societal resilience and national and regional disaster management capacities^55,56^.

The expected disaster response measures at the study target site should be carried out differently for each region (Fig. 7). For example, if the risk of forest fires is higher than that of drought, it is important to identify the main causes of forest fires and conduct long-term monitoring of prevention and dangerous areas. As a countermeasure, fire-resistant tree species can be mainly considered when planning afforestation^57^. According to Tng et al.^58^, giant eucalyptus and laban (Vitex pubescens), unlike other rainforest tree species, is a global pioneer who relies on fire for regeneration. If the risk of drought is higher than that of forest fires, the intensity, duration, spatial range, and rainfall of drought in vulnerable areas should be identified first^59,60^. Related measures include reducing the use of water for landscaping shrubs and trees, using non-vegetable water for irrigation during agriculture, cultivating drought or salt-resistant crops, and imposing excessive fees on consumers in case of water shortages. In addition, there is a way to increase water supply and supply pumps and pipes through water recycling. Similarly, if the risks of both drought and forest fires are similar or likely to occur in combination, it is desirable to plant plants or trees that may be more dominant in warm climates, such as the Giant Eucalyptus mentioned above. In addition, in terms of economic development and education, it is possible to consider providing incentives for agriculture and business diversification and civic education by disaster. In terms of health and nutrition, preparation plans are needed, such as establishing auxiliary programs for disaster victims and assigning light water for emergency evacuation in the event of a disaster. It is also expected that it will be necessary to conduct workshops for the general public based on various disaster topics that may occur due to climate change with technical support. It is also important to prevent drought and forest fires, but it is necessary to consider cases where related natural disasters occur frequently due to heat waves as climate change intensifies.

Forest fires in Kalimantan continue to increase from 2015 to 2019^14^. These fires can lead to droughts, and in the future, extreme climate change may limit current regulations and management methods^61^. The problem is that most Southeast Asian countries currently have low incentive to develop technologies to apply such knowledge and have no or ineffective regulatory instruments to strengthen such behavior^62^. The Association of Southeast Asian Nations (ASEAN) responded to fire smoke from forests and the wild through the establishment of a task force, the establishment of an action plan, and the negotiation of an agreement^61^. In most cases, these activities focus on symptoms and general cooperation on issues such as fire prevention, combat and monitoring^63,64^. However, Indonesia has not yet ratified a legally binding agreement in any case^14^.

Drought and forest fires can occur due to an increase in heat waves caused by climate change, and Indonesia is no exception. In particular, Kalimantan in Indonesia is predominantly composed of peatlands^15^. These peatlands are highly prone to fires during drought periods, and their extensive coverage can lead to large-scale fire incidents^16^. Numerous prior studies have emphasized the risks associated with peatland fires and the importance of their mitigation and recovery^65–67^. For instance, it has been documented that major fire incidents predominantly occur in peatland areas concentrated in the central and southern parts of Kalimantan^68–70^. Notably, the exacerbation of both climate change impacts and direct human interventions, such as urban development and plantation activities, has left limited margin for mitigation and has expanded the potential for greater damage.

Drought and forest fires can cause crop damage, resulting in a food crisis due to a sharp drop in agricultural production, and the ecosystem of plants and animals can be destroyed. In addition, smoke and soot generated during forest fires leave the region and deviate from the national boundaries, which can no longer be said to be a problem only for one country. In addition, the risk is expected to increase as the two disasters can occur in conjunction with each other. The main content of IPCC Working Group AR6^71^ states that heat waves and droughts cause forest fires, but according to Littell et al.^72^, forest fires also cause droughts. First, soot from fire contains organic carbon derived from plant tissue and fine dust (< 2.5 lm) formed through incomplete combustion^72^, which can affect precipitation along with local-scale cloud cover and suggest that forest fires can enhance or prolong drought events^73^. In addition, carbon dioxide emissions from forest fires into the atmosphere result in increased solar radiation absorbed on the surface, resulting in drought^74^. Conversely, when drought can affect forest fires, the likelihood of forest fires increases as drought reduces moisture stored in vegetation^75,76^. The slower the drought recovery, the greater the risk of forest fires, and this probability was investigated to vary depending on each climate and ecological condition^77,78^. As a result, if the severity and frequency of forest fires and droughts increase due to climate change and heat waves, ecosystem functions and structures can change rapidly^79^. Government efforts and budgets for response and recovery are expected to pose greater challenges after 2040~2059 and 2080~2099 depending on the RCP-SSP scenario, as shown in this study, suggesting that past disaster management approaches need to be reconsidered.

The MH risk map reviewed in this study was able to efficiently predict the risk probability area by analyzing 11 factors such as climate, infrastructure, topography, and land use factors. These results can be extended for use in the global disaster risk prevention and response phase. As a limitation of this study, only the risks of stages 4 and 5 were considered among the risks divided into 5 stages when preparing a MH map. To compensate for this, further research is expected to be needed on the basis for dividing this risk stage. It is considered that it may vary from region to country as a relative thing that divides these risk stages. Therefore, in future studies, we expected that it is necessary to establish their own risk level for each region through regional case studies.

## Conclusions

This research used a machine learning model to assess the geographic distribution of risk from MH in Kalimantan Island, Indonesia. Mountainous and island regions are prone to exposure to MH, and the locations within them are confronted by a broad range of natural hazards. The most critical problem for most decision-makers and natural resource managers is the identification of high-risk regions. In this respect, we provided a MH risk plan for the research area’s risks of forest fires and drought.

The study’s findings were predicted to be impacted by the risks of a 22.6% drought and a 21.7% forest fire, and 2.6% of the locations examined were predicted to be affected by both hazards. These risks were anticipated to increase in both RCP-SSP2-4.5 and RCP-SSP5-8.5. West Kalimantan had the highest risk of forest fires (33.02%), and South Kalimantan had the highest risk of droughts (67.3%) and both hazards (7.1%) for out of the five provinces in the study area. This was shown in the worst-case scenario, RCP-SSP5-8.52080, with the West Kalimantan forest fires rising to 33.51%, the South Kalimantan drought of 74.01%, and both disasters rising to 16.34%. In the future, climate change and heat waves may increase the severity and frequency of forest fires and droughts. Therefore, this study suggests that past hazard risk management approaches need to be reconsidered to prepare for the rapid transformation of ecosystem functions and structures due to increasing hazards.

RF model produced forecasts with respectable levels of precision. As a result, there is high trust in these findings, which can be applied to future research to investigate the geographic patterns of risks from MH and to provide crucial data for preemptive management and hazard mitigation.

### Supplementary Information

Supplementary Figure 1.

## Data Availability

The datasets used and/or analysed during the current study available from the corresponding author on reasonable request.
