# Supplementary material for: Multi-hazard exposure mapping under climate crisis using random forest algorithm for the Kalimantan Islands, Indonesia
Source: Sci Rep. 2023 Aug 18;13:13472. doi: 10.1038/s41598-023-40106-8 (PMC10439166; doi:10.1038/s41598-023-40106-8)
Supplement: Supplementary file 1 — Supplementary Figure 1. [file 41598_2023_40106_MOESM1_ESM.pdf]

Supplementary

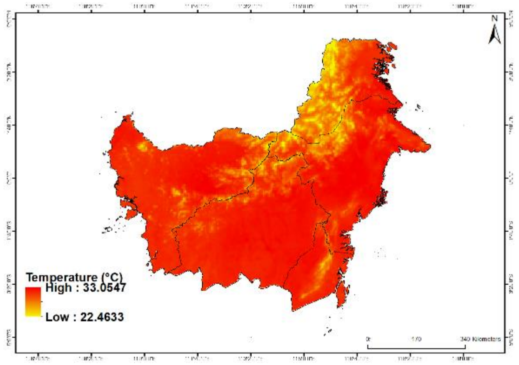

Temperature

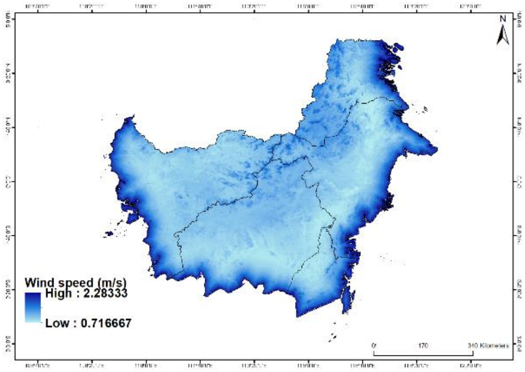

Wind speed

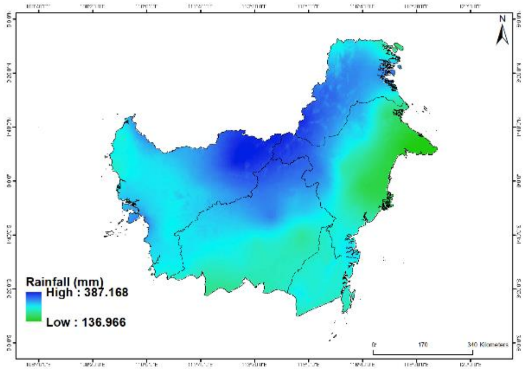

Rainfall

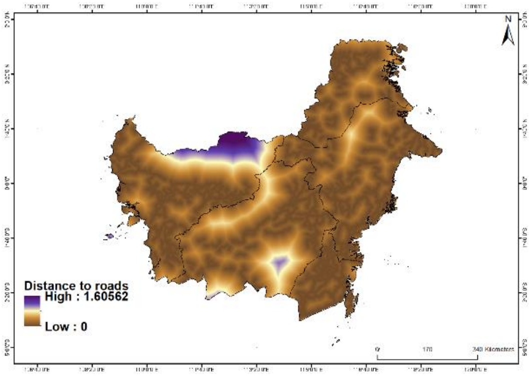

Distance to roads

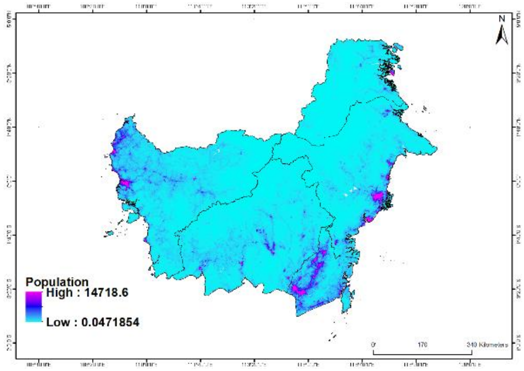

Population

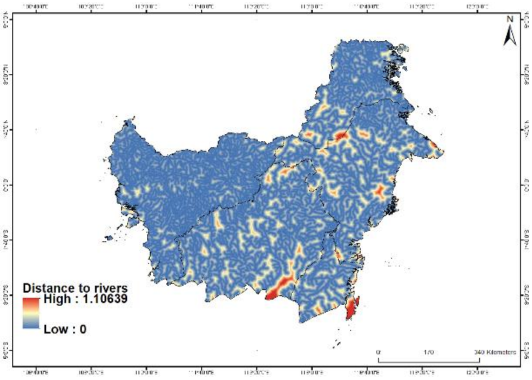

Distance to rivers

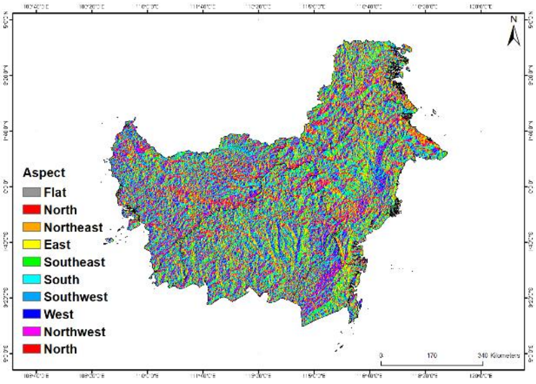

Aspect

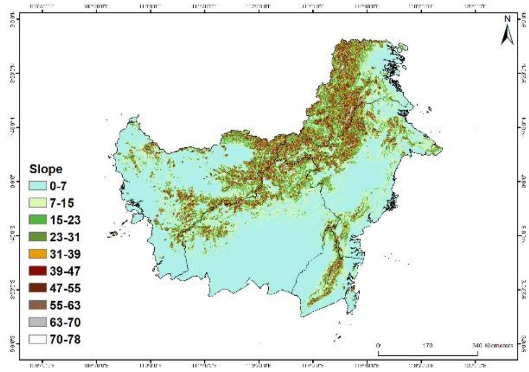

Slope

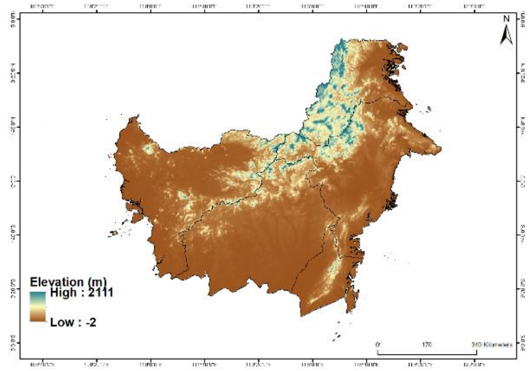

Elevation

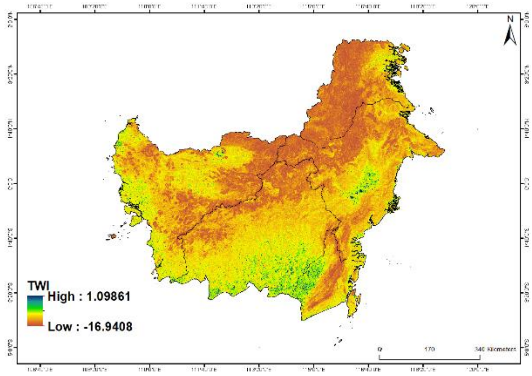

TWI

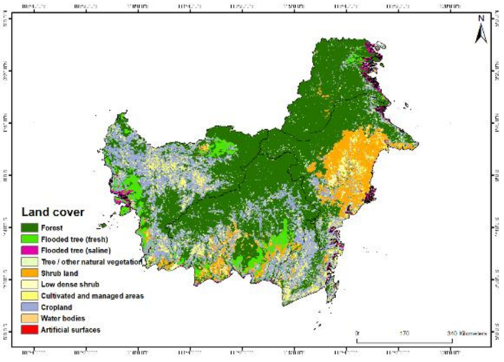

Land cover

Fig. 1. Multi-hazard conditioning factors in Kalimantan
